# Machine Learning Models to Predict Childhood and Adolescent Obesity: A Review

**DOI:** 10.3390/nu12082466

**Published:** 2020-08-16

**Authors:** Gonzalo Colmenarejo

**Affiliations:** Biostatistics and Bioinformatics Unit, IMDEA Food, CEI UAM+CSIC, E28049 Madrid, Spain; gonzalo.colmenarejo@imdea.org

**Keywords:** childhood obesity, obesity, overweight, machine learning, deep learning, statistical models, data science, BMI

## Abstract

The prevalence of childhood and adolescence overweight an obesity is raising at an alarming rate in many countries. This poses a serious threat to the current and near-future health systems, given the association of these conditions with different comorbidities (cardiovascular diseases, type II diabetes, and metabolic syndrome) and even death. In order to design appropriate strategies for its prevention, as well as understand its origins, the development of predictive models for childhood/adolescent overweight/obesity and related outcomes is of extreme value. Obesity has a complex etiology, and in the case of childhood and adolescence obesity, this etiology includes also specific factors like (pre)-gestational ones; weaning; and the huge anthropometric, metabolic, and hormonal changes that during this period the body suffers. In this way, Machine Learning models are becoming extremely useful tools in this area, given their excellent predictive power; ability to model complex, nonlinear relationships between variables; and capacity to deal with high-dimensional data typical in this area. This is especially important given the recent appearance of large repositories of Electronic Health Records (EHR) that allow the development of models using datasets with many instances and predictor variables, from which Deep Learning variants can generate extremely accurate predictions. In the current work, the area of Machine Learning models to predict childhood and adolescent obesity and related outcomes is comprehensively and critically reviewed, including the latest ones using Deep Learning with EHR. These models are compared with the traditional statistical ones that used mainly logistic regression. The main features and applications appearing from these models are described, and the future opportunities are discussed.

## 1. Introduction

Obesity and overweight prevalence among children and adolescents has increased to a large extent during the last four decades [1,2]. For instance, the prevalence of overweight and obese children and adolescents between 5 and 19 years has soared from about 4% in 1975 to 18% in 2016 [3]. This increase is especially dramatic in developing countries [4], while in developed countries it seems to be slowing down and affects mainly the low-income sub-populations [5]. In absolute numbers, it is currently estimated that about 38 million children under the age of 5 are overweight or obese, while about 340 million children and adolescents aged 5–19 years are overweight or obese [3].

This large prevalence poses a threat to the current and future health systems. Childhood and adolescent obesity is related to different comorbidities during this age [6,7,8,9,10], as well as to a lower quality of life [11], but, in addition, it is also associated to *adult* comorbidities, like metabolic syndrome and diabetes [12], cardiovascular risk [13,14], and death [15,16]. This is probably due to the difficulty in its eradication once it is established, justifying the adoption of childhood preventive measures, rather than therapeutic ones [9].

Obesity, that is, excess adipose tissue in the body [17], has a complex, multifactorial etiology. Among the factors involved in its development, the most important ones are genetics, physical activity, sedentary lifestyle, diet, etc. [18] In addition, obesity has additional complications for its analysis during childhood and adolescence. This is largely due to the huge changes in height and weight during this period. If we measure the Body Mass Index (BMI) through it, we see a pattern of an initial increase until reaching a first peak at about 1 year, followed by a decrease up to the age of about 6 years, where it starts to rise again (the so-called *adipose rebound*) [18]. So big are these changes that there is no universal consensus in the definitions of “overweight” and “obese” based on BMI at these ages [17], and in most cases, they are defined using sex-, age- and population-specific percentiles, normally ≥ 85th percentile for overweight, and ≥ 95th percentile for obese, as will be discussed in Section 4 in detail. (It must be noted that in this Review we use the concept “obesity” in two ways: one is as excess adipose tissue in the body in general, and the other is a BMI-based category to classify individuals, normally for adults BMI ≥ 30 kg/m^2^ and for children with multiple definitions as described in the text.)

Therefore, during this period, there happen large metabolic and hormonal changes that largely influence the adiposity at different ages. On top of that, there is still a large influence of specific pre-gestational and gestational factors, especially during early childhood, that have a large impact at these ages. The additional risk factors for obesity in childhood-adolescence have been reviewed recently [18,19]. Some of the most outstanding ones are parent’s BMI, gestational weight gain of the mother, gestational diabetes, maternal smoking, birth weight, rapid infant growth, and high protein and/or free sugars consumption. There are also psychological factors, especially during the adolescence period.

In order to prevent childhood and adolescent obesity, the development of predictive models to identify potential individuals of high risk is of great utility. This allows the focusing of preventive measures towards the high-risk subpopulation, allowing a more cost-effective and personalized approach to weight reduction interventions. In addition, the use of predictive models allows, by their analysis, to rank the different risk factors in order of importance, so that we can identify those that would be more effective in order to design these interventions. Moreover, the models can be used as simulation tools where “what-if” analyses can be conducted, by varying one or more predictor variables and seeing what would be the effect in obesity for particular sub-populations (defined by, e.g., sex, age, diet, etc.).

Given the large complexity of obesity, especially during the childhood and adolescence period, with a large number of multidomain influencing factors interacting in convoluted ways, traditional statistical methods like (generalized) linear models show limitations and have focused mainly in analyses with a reduced number of predictor variables and with limited predictive power. As we will see in Section 3, these models in most cases use more or less the same set of predictor variables transformed in one way or another and aggregated a linear functional form. Another limitation of these methods is their inability to deal with high-dimensional data, where the number of predictor variables (columns) is close or even much higher than that the number of dataset instances (rows), as they typically require many more instances than predictor variables in order to provide reliable inferences and avoid overfitting. Such situation makes them to need huge samples for they to be used with large sets of predictor variables, resulting in difficult practical implementations.

In this way, Machine Learning (ML) techniques are especially gifted modelling tools for these datasets, typically of high-dimensional nature and with complex relationships between many multidomain variables. This is due to their capacity to deal with high-dimensional data so that they can be applied to model relatively small datasets having large numbers of predictor variables and with reduced overfit. In addition, ML methods are able to find complex, nonlinear relationships between the predictor variables and these and the response variable or variables in an automated way, not requiring to manually predefine and test a large set of potential relationships between these variables. Therefore, the predictive capacity, ease of application, and robustness of these models for complex data far outclasses those of the traditional statistical models. This is even more in the case of the recent Deep Learning (DL) branch of ML, which can tap from huge datasets both in instances and predictor variables to obtain models with extremely good predictive capacities. DL methods, in addition, are able to directly use complex data like images, text, social media, time series, etc., avoiding the need of lengthy *feature engineering* processes, as we will see in Section 2. This is increasing dramatically the scope of data sources that can be used in this field, allowing to identify novel risk factors.

Given the above described advantages of ML over statistical methods for this problem, it is no surprise that ML have started to be used in the area. Thus, this paper attempts to conduct a critical and comprehensive review of the work done in ML models applied so far to the area of childhood and adolescent obesity. This will include a brief unbiased summary of each of the works available in the area to predict childhood or adolescent BMI and/or obesity/overweight with ML, followed by a thorough discussion of the collective patterns found, results obtained and novel risks factors identified, advantages and limitations of the approach, and future perspectives. The discussion will include also a comparison with the statistical models of the same outcomes, which will have been briefly reviewed previously. In addition, models to predict related outcomes (e.g., success of weight decreasing therapies, social obesogenic environments, pediatric attention to obesity, etc.) will also be reviewed, as they are of increasing interest especially in the area of preventive interventions. We will see that this is a new field that has experienced a recent explosion, especially during the last five years, mainly through the use of massive databases of Electronic Health Records (EHR) and the application for the first time of DL techniques, which is starting to allow a more systematic analysis of large cohorts with many multidomain predictor variables and the introduction of complex data sources as predictors. As the reader will see, this is also a very heterogeneous field, both in terms of type of model (cross-sectional, longitudinal), label predicted by the model (obesity, overweight, success of obesity therapies, pediatric attention to obesity, etc.), aim of the predictions (explanatory, predictive, and simulation), and application of the model (prediction of risk subpopulation, optimization of obesity therapy, suggestion of novel therapeutic approaches, etc.), further extending those typical of statistical models. It is expected to provide an updated view of the field to researchers within multiple disciplines and interests: statisticians, engineers, data scientists, epidemiologists, pediatricians, nurses, and nutritionists.

The article will be organized as follows: after this Introduction, first, a summary of the ML field will be conducted in order to provide some basic knowledge for readers not experts in the field, trying to make the work as much self-contained as possible; second, the procedure to search and select the reviewed works will be described; third, the statistical models in the childhood/adolescence obesity area will be reviewed, in order to set a comparison point with the ML models; fourth, ML models targeted to the prediction of BMI or categorized versions of BMI will be reviewed; fifth, ML models targeted to the prediction of related outcomes will be reviewed; sixth, a final wrap-up discussion of the main patterns in the models summarized will close the paper.

## 2. Basic Concepts in Machine Learning

*Machine Learning* (ML) exploded in the 90s of last century as a new field of data analysis at the interface between Statistics and Artificial Intelligence. Although the initial concepts like Rosenblatt’s perceptron [20] (a basic, 1-layer artificial neural network to perform binary classification), Naïve Bayes [21], Decision Trees [21], and k-Nearest Neighbors date back to the 50s–60s of the 20th century, it was during the last decade of it when the field started to enter into full maturity and be massively applied. This happened with the appearance of multi-layer neural networks, thanks to the invention of the *backpropagation* training algorithm [22], as well as other ML paradigms like Support Vector Machines [23] and, in the first decade of the 21st century, Random Forests [24] and Gradient Boosting Machines [25]. This emergence has been fostered by the confluence of CPU miniaturization and cheapening, massive accessibility of computational capacity, and the development of completely new ideas for statistical modeling.

This explosion has been followed, in the second decade of the 21st century, by the one of *Deep Learning* (DL). DL is an outgrowth from ML that comprises mainly artificial neural networks of very large numbers of layers (the term “deep” comes from here), together with specialized layers, like *convolutional* and *recurrent* ones, and additional adaptations to allow the training of these huge neural networks: non-saturable activation functions; new weight initialization schemes; faster optimizers; and the training of the network in small, random batches of the data (the so-called *mini-batch* training). The DL models contain typically millions of training parameters. The specialized layers find directly from complex data like images, sounds, texts, music, etc., patterns (“feature maps”) that are fed into multi-layer fully-connected perceptrons, allowing the direct modeling of this complex data, without the need of manually generating compressed representations of these data, the so-called “feature engineering”.

Again, DL has benefited from an additional increase of computational power easily accessible, mainly though both the use of GPUs instead of CPUs, and of cloud computing, as well as the availability of huge public datasets (e.g., YouTube, San Bruno, CA, USA; Wikipedia, Facebook, Menlo Park, CA, USA; etc.) and open competitions (Kaggle, San Francisco, CA, USA, etc.).

Generally speaking, ML has put more emphasis in *prediction* rather than *testing of a predefined hypothesis* like traditional statistical models, where the emphasis is more in inference. In the same way, the focus is more in a practical, engineering-oriented approach rather than on a rigorous theoretical background. ML can be defined as a set of algorithms that *automatically learn simplified representations of the data*. For example, we can present the ML algorithm with a set of data instances, like pictures of animals, together with a label for the species present in each picture. The algorithm would then be *trained* by automatically learning some abstract internal rules to associate each image to each label, by minimizing some kind of measure of the prediction error or *loss*. When presented with new pictures, the algorithm would then be able to assign a label (species name) to each of them.

ML models are able to cope with very complex datasets, even those with many more predictor variables than instances (*high-dimensional datasets*). For this reason, they tend to be more difficult to interpret (“black-box” type of models), although as we will see later, new techniques have been developed to facilitate understanding the inner working of the model.

From our purposes in this Review, we can talk about two main groups of ML models: *supervised* and *unsupervised*. Supervised models are those that use datasets comprising both a set of *predictor variables* and one or more *target variables* or *labels*. The model would then be trained to be able to predict the label(s) from new instances of the predictor variables: for instance, to predict if a child will be obese or not from his age, sex, parent’s BMI, and food consumption. The other type of ML models, *unsupervised ones*, attempt to find, without the use of labels, transformations of the input data with easier visualization, less noise, etc., or try to identify groups in the data. These techniques include *Dimensionality Reduction* and *Clustering* techniques.

Within the area of supervised models, which are the ones we will see in the Review, there are two main groups: *classification* models, those where the predicted label is a categorical one (e.g., obese child yes or not), and *regression* models, those where the label is a numeric one (e.g., BMI).

**Figure 1:**
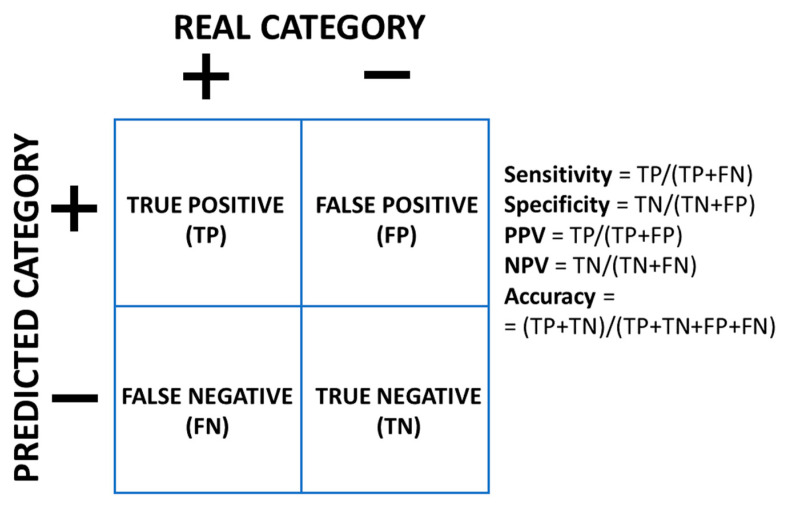
Measures of the performance of a binary classifier. Class labels are “+” and “−“. Predicted category by the model is represented vs the real category, for all the possible situations.

The most important type of classification models is *binary classification*, where the label has only two categories, for instance “+” and “−”. In this case, the model frequently outputs a probability *p* of one of the two classes (e.g., “+”; the probability of the alternate class “−” would be 1 − *p*). Once we define a threshold *t* for this probability, if *p ≥ t* for a new instance, we would assign the category “+” to that instance; if, on the contrary, *p < t*, we would assign the category “−”. At this point, several concepts are used to characterize the performance of the model (Figure 1), depending on whether the real category is “+” or “−“, and whether the predicted category is “+” or “−“.

*Sensitivity* (or *recall*) is the proportion of real positives that are predicted as positives. *Specificity* is the proportion of real negatives that are predicted as negatives. *Positive Predictive Value* (PPV), or *precision*, is the proportion of predicted positives that are real positives, and *Negative Predictive Value* (NPV) is the proportion of predicted negatives that are real negatives. *Accuracy* is the total proportion of correct predictions of all the predicted data.

A perfect model would have all these measures equal to 1. Obviously, this is almost never the case, and we have to cope with some proportion of errors. We can choose the threshold *t* so that it optimizes the purpose of our model. For example, if we are mainly interested in identifying as many real positives (e.g., future obese children) as possible, in order to apply to them some preventive weight-loss treatment, we would select a lower *t* and thus increase the sensitivity, even at the cost of increasing the false positives and, therefore, decreasing the specificity and the PPV. This approach would reach a point where we would identify so many false positives that would result in a prohibitive cost for treating many unnecessary cases or, if applying the treatment to a future normal-weight child has a negative effect, an unnecessary harm to too many members of our population. Alternatively, if we are more interested in finding a sample of children most of whom will be obese in the future, even if it is small (e.g., we can use it later for genotyping purposes), we would be more interested in optimizing the PPV; in this case, we would use a larger *t*, therefore increasing the false negatives. This would result in a decreased sensitivity and NPV. Again, we cannot increase *t* indefinitely, because there will be a point where the sample would be so small that would become useless. Therefore, there is always a balance between the cost and benefit, not just from the statistical point of view but also from the practical application of the model, which must be taken into consideration when optimizing the threshold of the model.

**Figure 2:**
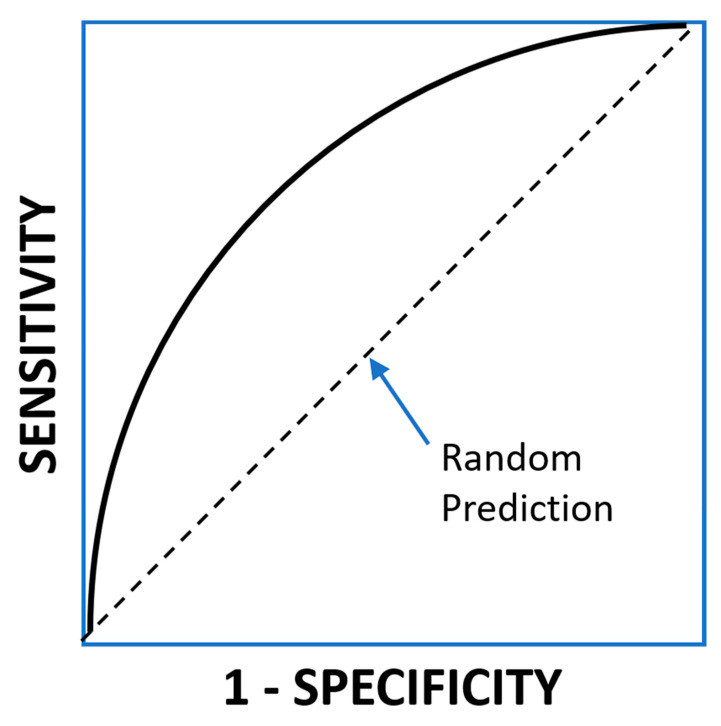
ROC curve of a binary classifier.

In order to characterize the discriminative capacity of the model, before selecting *t*, it is customary to use Receiver Operating Characteristic (ROC) curves. In this curve, the sensitivity is plotted against 1-specificity for all the values of the threshold (Figure 2).

For a random classifier, the curve will be a diagonal going from (0, 0) to (1, 1). For a perfect classifier, the curve would go from (0, 0) to (0, 1) and then to (1, 1). Intermediate classifiers would have a curve in between these two extremes. A frequent measure of the discriminatory power of the classifier is the area under the curve of the ROC curve (AUCROC). A random classifier has an AUCROC of 0.5, and a perfect classifier has an AUCROC of 1. Real-life classifiers would have values in between, the better the closer to 1. The AUCROC equals the so-called *concordance index* or *c-index*. 

When the classifier predicts a multi-class label, that is, with more than two classes, a measure of the prediction performance is the accuracy, defined above as the percentage of instances for which the label is predicted correctly. Another measure is the *categorical cross-entropy*. For a prediction instance *i* and an M-category label, it is defined as the Equation (1)

(1) $$-\overset{M}{\underset{j=1}{\sum }}I_{ij}logP_{ij}$$

where *I_ij_* is an indicator variable that is 0 if the predicted class *j* of instance *i* is not correct and 1 if it is. *P_ij_* is the predicted probability for class *j* on the new instance *i*. For *n* predicted instances, the categorical cross-entropy would be the sum of each of the instances categorical cross-entropies. Therefore, it basically measures the match between the predicted probabilities for the different classes with the observed frequencies. The better the agreement between predicted and actual labels, the smaller the categorical cross-entropy, thus being an *error* or *loss* function that is minimized as the model is trained with the training data (in the case of accuracy, it would be maximized). For binary classifiers, the cross-entropy formula simplifies to *M* = 2, and we have the *binary cross-entropy*.

When we deal with regression, common measures of the error or loss are the *Mean Squared Error* (MSE, the Equation (2)):

(2) $$MSE=\frac{1}{n}\overset{n}{\underset{i=1}{\sum }}{\left(y_{i}-\hat{y_{i}}\right)}^{2}\;$$

where *n* is the number of predicted instances, *y_i_* is the actual continuous label for instance *i*, and $\hat{y_{i}}$ is the predicted value of the label for that instance. Another is the *Mean Absolute Error* (MAE, the Equation (3)):

(3) $$MAE=\;\frac{1}{n}\overset{n}{\underset{i=1}{\sum }}\left|y_{i}-\hat{y_{i}}\right|$$

where |.| means absolute value.

When training a model with training data, there is a risk that the model learns too many details of the latter, which makes it perform worse when presented with new data. In this case, we say that the model is *overfit*. Normally, all the models we fit will fit better the training data than new datasets. Therefore, in order to assess the *practical* prediction performance of a model, we need to *validate* it with new data. We will see that there are different approaches for validation of models, which can be divided in two groups: *internal validation* methods and *external validation* methods.

The main feature of internal validation methods is that we resample several times from the whole dataset, fit a new model with the resample, and evaluate the model with the instances left out from the resample. From this repeated resampling, model fit, and evaluation, we get an estimation of the predictive performance of the modeling *process* with new data, although we do not really test a final model with new data. Internal validations are used normally when the data is scarce, so it is very difficult to obtain a new data set to externally validate the model.

There are two main general approaches for internal validation: *cross-validation* and *bootstrap*. In the former case, in its *k-fold* version, what we do is divide the total sample into *k* random subsets (“folds”; as for *k*, normally 5 or 10 is used). Then what we do is, for each fold, validate with this fold a model fitted with the *k* − 1 remaining folds. The estimated validation measure of the performance of the model (accuracy, cross-entropy, MSE, etc.) will be the average of the performances of the *k* models fitted with each *k* subsamples, each having with *k*−1 folds and evaluated in the corresponding hold-out fold.

Cross-validation schemes can also be used to estimate *hyperparameters* of our model (e.g., number of nearest neighbors in the k-Nearest Neighbors method, see below). What we do then is perform the cross-validation with a double loop of folds; in one loop, we vary the hyperparameter among several options, and in the other, we estimate the validation performance within each hyperparameter selection. We will select the hyperparameter value that optimizes the cross-validation performance estimate, and at the same time that performance will serve as estimate of the external performance of the model (fitted with that optimal hyperparameter value).

It must be taken into account that the models fitted with cross-validation use a smaller dataset than the whole dataset, so this can be a source of error of estimation of the performance. The other approach for internal validation, bootstrap, avoids this issue by generating repeatedly samples of the same size of the original one by sampling with replacement (allowing randomly repeated instances). The model is refitted for each of these random samples and then evaluated in both that sample and in the original sample or the left-out instances. By averaging the difference between the training performance in each sample and the performance in the original sample, we get an estimation of the so-called *optimism* in the training performance. Then, we would derive the model with the whole dataset, evaluate its performance, and correct it by the estimated optimism.

We see that in both cross-validation and bootstrap, we do not make a real evaluation of the external performance of the model but rather make an estimation of it from data that is used at the end in the derivation of the final model. The alternative is to use an *external validation sample*. This is data that is not used in the derivation of the model and is only used for validating the model. A simple approach here is to randomly split the original sample into a training dataset (e.g., 60–80% of the data) to fit the model with it and then a validation/testing dataset (40–20% of the data) to evaluate external performance. This has two drawbacks when compared with internal validation methods: on one hand, we miss some of the data in the model derivation; on the other hand, we make the estimation of the external performance with a normally small dataset, which would result in an estimate with high variance (depending on how “lucky” we are in the random split, we can have very different estimates). This is not an issue if we have a very large dataset, and the validation set is quite large. However, in case we have a small dataset, it is preferable to use internal validation measures, despite being a bit more optimistic than external validations.

In addition, the random split approach has an additional problem in that both the training and the validation datasets come from the same sample, and thus, it is likely that they are very similar, a situation that quite possibly does not to occur when using the model in real life. There are ways to avoid this issue, like clustering the original sample and then generating training and validation datasets from different clusters. Another approach is to train the model with one dataset and then validate it with a different dataset, e.g., a posterior in time dataset, a dataset from another country, etc. This is a more demanding comparison but is probably the closest to the real performance of the model in production. Obviously, this approach is very expensive in terms of datasets, so it is only available in a reduced number of situations.

We will finish this section by briefly describing the ML models we will see in the Review.

### 2.1. Naïve Bayes (NB)

This method uses Bayes rule together with the approximation of conditional independence of predictor variables given the response class. Bayes rule establishes the posterior probability of the target variable *y* (label) taking the value *j*, conditioned to the predictor variables *x*_1,_ …, *x*_*n*_ (the Equation (4))*:*

(4) $$P\left(y=j|x_{1},\ldots ,x_{n}\right)=\frac{P\left(x_{1},\ldots ,x_{n}|y=j\right)P\left(y=j\right)}{P\left(x_{1},\ldots ,x_{n}\right)}$$

where P(y=j) is the prior probability of *y* taking the value *j***,** $P(x_{1},\ldots ,x_{n}|y=j)$ is the posterior probability of the predictor variables conditioned to *y* taking the value *j*, and $P\left(x_{1},\ldots ,x_{n}\right)$ are the prior probabilities of the predictor variables. These prior and conditional probabilities can be estimated from the respective empirical frequencies when the predictor variables are categorical. When they are continuous, they can be approximated by different kernel functions. When the independence approximation is applied in NB, this simplifies largely (the Equation (5)):

(5) $$P\left(y=j|x_{1},\ldots ,x_{n}\right)=\frac{P\left(y=j\right)\prod_{1}^{n}P(x_{i}|y=j)}{P\left(x_{1},\ldots ,x_{n}\right)}$$

The predicted class for a set of *x*_1_, …, *x_n_* predictor values will be the one that maximizes the productory above, since the other factors are constant.

### 2.2. k-Nearest Neighbors (kNN)

The idea of this method is quite simple: For a new instance with predictor variables *x*_1_, …, *x_n_*_,_ assign the label most frequent between the *k* instances in the training data with predictor variables less distant (more similar, the k-*nearest neighbors*) to the new instance predictor variables. This is called the *majority voting* class assignment. When the label is a continuous one (regression), the predicted value is the (weighted) average of the labels of the k-nearest neighbors. In order to measure the distance between sets of predictor variables, different metrics can be used. Probably the most frequent is the Euclidean one. The value of *k* can be quite variable and depends heavily on the dataset. It can be obtained through cross-validation techniques.

### 2.3. Decision Trees (DT)

**Figure 3:**
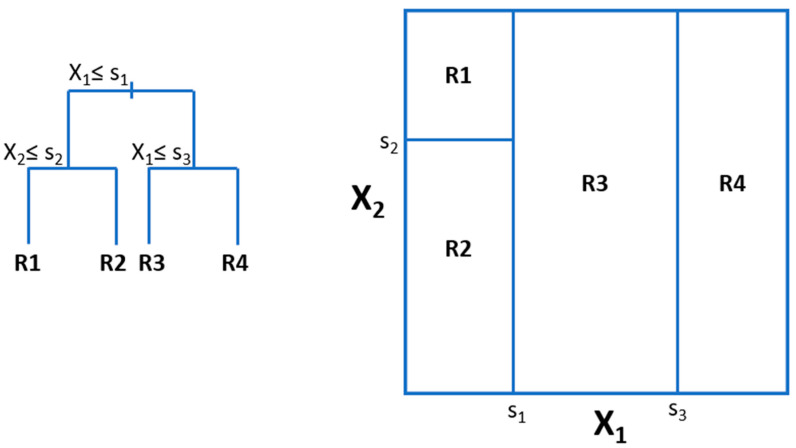
Depiction of Decision Tree for two variables, X_1_ and X_2._ R1_,_ R2, R3, and R4 are partitions generated by the splits s_1_, s_2_, and s_3_. The labels for the partitions would be a function of the labels of the instances in each partition in the training set.

This method can be used for both regression and classification. The idea here is to generate rectangular partitions of the space of predictor variables, by successive splitting the data by (usually binary) splits in one variable that optimize some loss function (e.g., minimization of MSE for regression). At the end, the label we assign to each partition is one function of the labels of the data instances belonging to each partition, e.g., its mean, or the majority voting class. Then, for new instances, we will find the partition it belongs to and assign the label that corresponds to that partition. A simple schema with only two predictor variables is depicted in Figure 3.

Obviously, to grow a tree can become a very complicated task, given the combinatorial number of possible splits and variable sequences that can be created. Therefore, simplified algorithms for generating the tree have been devised. There are different ones, depending on the criteria for split, the selection of variables at each split, and the pruning of terminal nodes. These are CART [26], on one hand, and ID3 [27], which evolved to C4.5 (also called J48 in Weka’s Java implementation) and later to C5.0. There is also the CHAID [28] algorithm, based on statistical tests and allowing non-binary splits.

The advantage of DT is the ease of interpretation, which can be aided by graphical displays; however, they are known for the high variance of their predictions, such that little variations of the dataset can result in very different trees and predictions.

### 2.4. Support Vector Machines (SVM)

**Figure 4:**
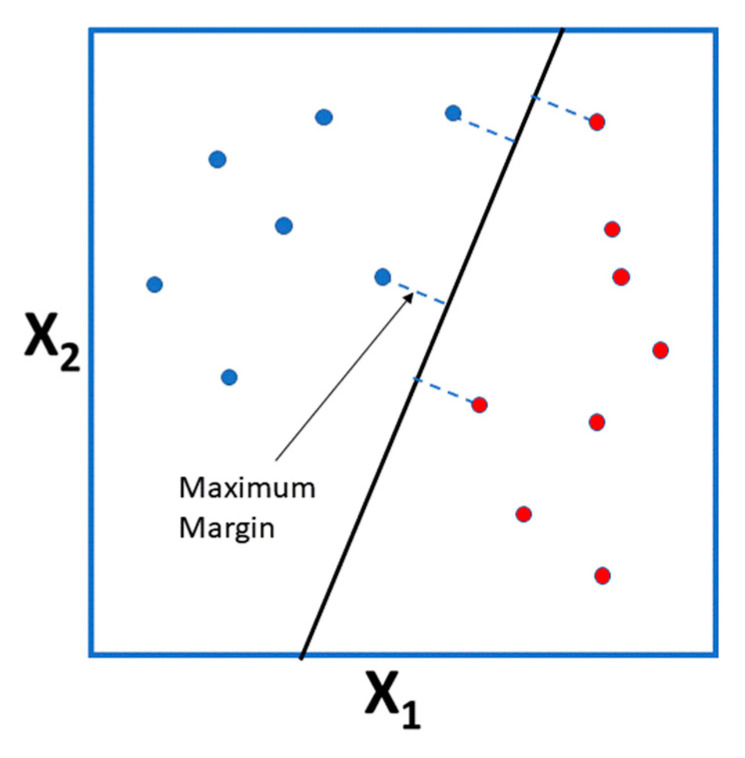
Maximum margin hyperplane for a predictor space of two variables. Two categories are perfectly classified by this hyperplane. The hashed lines indicate the maximum margin to the training set, obtained with this particular hyperplane. Training instances are presented as points in the plane, blue points corresponding to class “+” and red points to “−”. The points located at a maximum margin to the hyperplane are the *support vectors*, since the plane only depends on these points of the training set.

This method was initially developed as a binary classifier. The approach is to build a hyperplane from the predictor variables with maximal margin, so that one half of the predictor space would result in a “+” label and the other in a “−” label. By maximal margin is meant a hyperplane that has the largest distance to the training instances of the infinite possible hyperplanes or, more correctly, the farthest minimum perpendicular distance to the training instances (since the “margin” is the minimum distance the training set points have to the hyperplane). Figure 4 displays a dataset of two predictor variables and the corresponding maximal margin hyperplane for the training instances.

For new testing instances, we just need to find which side of the hyperplane the new point lies in order to predict a label for it.

As a matter of fact, it is usually the case that the points are not perfectly separable. Therefore, instead of a maximum margin hyperplane, a “soft” margin one is obtained, by allowing some latitude for misclassified points with some specific criterion. This also makes the method more robust against small modifications of the dataset. In addition, in many situations, the boundary regions between the two classes are not linear. In this case, what we do is include as predictor variables additional specialized functions of these variables and instances, the so-called *kernels*, such that the dataset becomes linearly separable. There is a variety of kernels yielding different types of the SVM method: linear, polynomial, radial, etc. It turns out also that the computation of the hyperplane only requires the closest points to the boundary, which are called the *support vectors*, making the computation much faster. From this, the method takes its name.

Later developments of the method allowed it to deal with multiclass classification as well as regression.

### 2.5. Random Forest (RF)

RF are an example of ensemble methods, where a model of higher quality is built by aggregating multiple models of lower quality. The prediction for new instances will be obtained by averaging the prediction of all the simple models in the case of regression or, for classification problems, by the majority voting. In this way, we make predictions much more robust, with much less variance and with higher accuracy.

In the case of RF, we use an ensemble of hundreds or thousands of DTs. In addition, these DTs are built without pruning so that they will have little error, although large variance. However, since we are averaging many of them, the final variance will also be low. These DTs are built from bootstrap samples of the original training dataset (this is called *bagging* or bootstrap averaging). Moreover, to decorrelate the trees, at each split in the tree, only a random subset of predictor variables is used. In this way, the reduction of variance by averaging the trees is more efficient.

A very interesting property of the RFs is that they incorporate internally a direct estimation of the external validation error. Since the DT models are derived using bootstrap samples, for each instance in the training set, there will be a set of trees (approximately B/3, where B is the number of trees since they are fit using bootstrap samples) that will have been derived without that instance. By averaging the difference in label prediction for that instance in these trees and its actual label, we would have what is called an out-of-bag (OOB) estimate for that instance. Averaging over all the instances, results in an estimate of the external performance of the RF without the need to use cross-validation or bootstrap.

RFs are a very powerful predictive method, both for regression and classification, and very robust irrespective of the type of datasets. The issue with them is the difficulty of interpretation (this is general for all the ensemble methods), since they contain many different and decorrelated DTs using different predictor variables. An approach used to analyze them is the so-called *variable importance techniques*. The idea here is to analyze the effect that each predictor has (on average over all the DTs) on the error of the RF. One approach is to calculate, for each predictor, what error reduction it has had each time it has been used in the trees. This is summed for all the trees, and the largest sum will correspond to the most important predictor as on average it has produced the largest reduction of errors in all the trees. Another approach uses permutation of the variables. For each tree, we have its OOB prediction accuracy after applying it to its OOB samples. After that, the *j*th variable is permuted and the OOB prediction is recalculated and subtracted from the previous one. This is averaged over all the trees. This is also repeated for all the predictor variables. We would then obtain a ranking of the variables, with those with the largest reduction of OOB performance being the top ranked.

### 2.6. Gradient Boosting Machines (GBM)

This is another ensemble method, but one that uses *boosting* instead of bagging. By *boosting*, it is meant the iterative improvement of a weak model by adding sequentially new models that improve the previous fit. In the case of gradient boosting machines, normally the models are DTs, and the improvement is done by fitting the new model to the residuals of the model so far or, more generally, to the gradient of the loss function we are using. Newer versions, like XGboost, use the second derivatives instead of the first ones, in order to improve speed and performance.

GBM, especially XGBoost, are currently the most used ML algorithms for models using numeric tabular data or (when modeling more complex data) feature pre-engineered data. For problems using complex data directly (e.g., computer vision, speech recognition, natural language processing, etc.) Deep Learning methods are used instead (see below).

As it happens with RF, the interpretation of these ensemble models is complicated. However, in the same way, techniques like variable importance can be used to facilitate interpretation.

### 2.7. Regularized Linear Models (LASSO)

When fitting linear models, the residuals of the least squares fit decrease as we add more predictor variables. However, if the number of instances *n* is not so much larger than the number *p* of predictor variables, the estimates of the least squares increase their variance as *p* becomes closer to *n* so that the model becomes overfit, and the external or test performance of the model decreases. In the case of *n* < *p*, the variance become infinite, no unique fit exists, and the method becomes useless. However, this situation of high dimensionality is very typical in ML datasets. One way to fix this problem is to shrink or *regularize* the estimates, so they remain small and with low variance, and in some cases, they even become zero. One approach to regularization is *ridge regression*, where all predictor variables are maintained, but their betas are kept small by restraining the sum of squared betas to be less or equal than a small value. Although this approach improves external performance of the model, it keeps an interpretation issue as no irrelevant variables are removed. An alternative approach is the LASSO, where the sum of the absolute value of the betas is restrained to being less or equal than a small value. This has the advantage of making some betas equal to zero, thus performing an effective selection of important variables.

### 2.8. Bayesian Networks (BN)

A Bayesian Network is a directed acyclic graph of nodes that correspond predictor variables, plus one or more nodes that represent the label(s). The directed edges between the nodes represent causal relationships between the variables, through conditional dependence, and Bayes rule is used to determine the probability of the different possible values of the labels conditioned to particular values of the predictor variables. Nodes not connected would be conditionally independent. There is a large set of techniques to infer the structure and parameters of the network.

### 2.9. Artificial Neural Networks (ANN)

**Figure 5:**
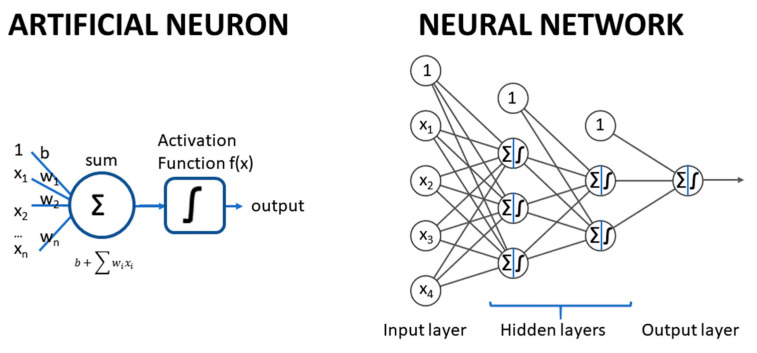
Typical structure of an artificial neuron and a fully connected feedforward neural network. The x_i_ are the predictor variables, the w_i_ are the weights and b is the biass.

ANNs are ML methods that mimic the structure and mechanism of the nervous system. They are composed of layers of artificial neurons, with connections between neurons in consecutive layers. Each artificial neuron is an abstract unit that applies a weighted sum of its numeric inputs plus a bias parameter, and the resulting sum is passed to a so-called “activation function” to generate a numeric output. The first layer corresponds to the input variables; these variables are used as inputs of the next layer neurons, where each of its neurons generate an output, which is then used as input of the next layer neurons, and so on. The last layer contains typically one single neuron for one label or more for multilabel models. Figure 5 displays a typical fully connected, feedforward ANN (multilayer perceptron).

The input layer contains the input variables (no transformation), while the last layer generates the output of the model and is called the output layer. In between, there are one or more layers, which are called “hidden” layers. Each neuron has a weight per input plus a bias parameter; all these weights and biases of all the neurons are the parameters of the network, which are optimized to minimize a loss function.

The first model or ANN was the perceptron by Rosenblat [20], which was designed as a one-neuron simple binary classifier after the mathematical neuron devised previously by McCulloh–Pitts [29]. The development of ANN to solve problems not linearly separable was allowed by the invention of the backpropagation algorithm [22], which allowed the training of multilayer perceptrons.

ANN became very popular in the 90s of last century, when they were amply used in many areas. At that time, they required feature engineering for many problems, and they were more or less abandoned in the first decade of the 21st century after the appearance of RF and GBM, since ANNs were slow to train, expensive computationally, and prone to overfitting. However, they have become very popular in the second decade of this century with the advent of the field of Deep Learning.

### 2.10. Deep Learning (DL)

The ML models we have seen so far have two main issues. On one hand, their performance in many cases shows *saturation*: This means that they reach a point when, irrespective of how big we grow the training set, the performance does not increase significantly. On the other hand, they work with numeric, tabular data, so they are unable to handle complex data like images, speech, text, etc. In order to model this type of data, it is required to convert it to numerical predictor variables in a very ad hoc and manual fashion. This is the so-called “feature engineering” problem. They are “shallow” methods, that is, unable to learn hierarchical representations of complex data.

These two problems are solved to a large extent with DL. DL consists mainly in ANNs with very large numbers of layers (that is the reason for the “Deep” in the name) and, therefore, huge numbers of training parameters. In this way, they are able to tap from huge datasets and increase steadily their performance without saturation.

On the other hand, some specialized layers have been developed that are able to automatically generate numerical representations (*feature maps*) of complex data. That is the case of *convolutional layers*, that are able to reformat tensor data of different dimensions. For example, in the case of 1D convolutional layers, they are able to find representations for serial data like text for language translation models; 2D convolutional layers are appropriate to model images like in computer vision models; while 3D layers can handle volumetric data like medical 3D images or video data.

Another specialized layers are the *recurrent layers*, where the output of the layer goes both to the next layer and to itself, allowing to find long-term and long-distance patterns by the use of ad hoc developed layers: Long Short-Term Memory (LSTM) [30] and Gated Recurrent Units (GRU) [31]. This type of layers is very appropriate also for serial data and is mostly used in natural language processing (NLP) applications.

Many of these specialized layers can be stacked sequentially and thus generate automatically hierarchies of representations with increasing levels of abstraction. This allows the model to learn very convoluted aspects of the data, which is not possible with the traditional ML methods. In addition, this hierarchical representation of the data can be applied to generate new specialized models with small datasets by reusing more general models fitted with much larger datasets. For example, we can develop one very efficient model to classify cats from a dataset of relatively few pictures of them by reusing some of the more abstract pre-fit layers of a more general model developed to classify animals from a huge dataset of pictures and adding to them some new layers that would be fit with the new small dataset of cat pictures. The previous layers would have learnt to identify the general shape of an animal, while the new layers would fit the specific features of cats. This is the process called *transfer learning*.

At the end of these layers, normally a multilayer perceptron is added to generate the output, whether numerical (regression) or categorical (classification).

DL is revolutionizing the ML area and is being applied in completely new fields, like drug discovery, music generation, self-driving cars, etc. They are also applied to biomedicine, [32,33] and as we will see, they have started to be used in the childhood obesity area.

After this summary of the main types of ML models, we proceed to describe the selection process of works reviewed in this paper.

## 3. Bibliographic Search and Selection of Works for Reviewing

An attempt was made for comprehensiveness in the bibliographic search, both in terms of time and publication media. Since the field of ML/DL applications is a very hot one, growing in an extremely fast way, it is not infrequent to find material published in congress proceedings, arXiv, etc. In addition, since this field shows a large interdisciplinarity, being at the interface between statistics, artificial intelligence, and biomedicine and including statisticians, engineers, pediatricians, nutritionists, and nurses in its research body, typical search engines used in biomedicine like Scopus, PubMed, etc., were not used in the search, as they missed many of the available references. Instead, Google Scholar was used for the bibliographic search. The search was performed by iteratively querying the engine with appropriate keywords in order to find papers that applied ML to predict childhood/adolescent obesity/overweight (e.g., childhood OR child OR adolescent AND machine learning OR data mining, etc.), extracting the matches and matching references in the corresponding bibliographies, and updating the queries after the titles of the matching references if necessary. This procedure was repeated until no new matches were obtained. Concept papers not applied to a particular dataset were not included.

On next section, the most outstanding statistical models in the literature to predict childhood and adolescent obesity will be briefly reviewed. These will be used as comparison point to the ML models, that will be reviewed afterwards.

## 4. Statistical Models to Predict Childhood/Adolescent Obesity

There has been a lot of work performed to derive statistical models to predict childhood/adolescent obesity [34,35,36,37,38,39,40,41,42,43,44,45,46,47]. Although in principle it is advisable not to categorize variables when deriving models, whether predictor or target ones, given that the process results in a loss of information, most of the work in this area has focused on classification models for overweight, obesity, or combinations of them. The reason is obviously that most of the clinical interest is in detecting the conditions that can lead to pathological complications, and these are overweight and obesity, not BMI or similar endpoints in general. Another pathological nutritional status is undernutrition, but it is outside the scope of this Review. In the case of children and adolescents, given the large variability of both height and weight during this period of life, there is no general consensus in the definitions of overweight and obese [17], and the single-cutoff definitions used with adults, namely BMI ≥ 25 kg/m^2^ for overweight and BMI ≥ 30 kg/m^2^ for obesity, following the WHO definition, [3] are not valid. Instead, the common practice in the case of children/adolescents is to refer the BMI to an age- and sex-based (in some cases ethnicity too) distribution of BMI of the population at hand. The most common criterion is to define as overweight a child whose BMI is equal or above the 85th percentile for that sex and age and as obese a child whose BMI is equal or above the 95th percentile. As we will see, in most of the cases, these percentiles are obtained from the Centers of Disease Control (CDC) data if the sample is from the US [48] or alternatively from WHO growth charts [3], charts from the International Obesity Task Force (IOTF) [49], or growth charts from samples in other countries (e.g., UK90 for UK [50]).

Previous recent reviews in the area are those of Butler et al. [51], Ziauddeen et al. [52], and Butler et al. [53], all of them from 2018. Here, we will briefly review all the works found there and additional ones, in order to provide some sort of baseline predictive models to compare with the ML ones. A total of 14 papers have been found. Table 1 summarizes the main features of these models.

The most used tool to develop the statistical models is logistic regression, which is applied to predict binary outcomes. Here, a linear equation is used to predict the log-odds of a binary variable displaying one of its two alternative categories vs. the other, like being obese or being overweight vs. normal weight. This is the case of all the works but two exceptions. One is the work by Cortés-Martín et al. [47], where proportional-odds ordinal logistic regression is used, which is an statistical model appropriate to predict ordinal variables. In this case, the predicted outcome was the three ordered categories of BMI, namely normal weight vs. overweight vs. obese for children and adolescents (5–17 years). The other case is the work by Mayr et al. [38] where the authors use quantile regression with boosting to derive prediction intervals (which are at the end quantiles of the BMI for future observations) for BMI at different ages in childhood.

In addition, in the paper by Pei et al. [40], the standardized BMI at 5 years was also predicted by means of linear regression, together with obesity at 10 years with logistic regression. Moreover, in the paper by Druet et al. [35], a metanalysis is performed from the odds-ratios obtained from several logistic regressions for 10 different cohorts of variable nationality to estimate an odds-ratio for childhood obesity as a function of the 0–1 year weight gain standard deviation score (SDS).

The rest of the papers aim at the prediction of overweight or obesity at one or several ages or a range of ages exclusively by means of logistic regression. Papers focused on the prediction of overweight are those of Steur et al. [34] (at 8 years), Weng et al. [41] (3 years), Graversen et al. [43] (at adolescence), and Redsell et al. [46] (5 years). Papers focused on the prediction of obesity are those of Druet et al. [35] (7–14 years), Levine et al. [36] (5 years, stratified by sex), Manios et al. [39,44] (9–13 years), Pei et al. [40] (10 years, as said before), Santorelli et al. [42] (2 years) and Robson et al. [45] (5 years). In the paper by Morandi et al. [37] both endpoints are predicted: overweight and obesity at both 7 and 16 years; in addition, predictions are made for *persistent* overweight and obesity, that is, overweight and obesity at *both* 7 and 16 years. By considering the definition of overweight and/or obesity in these works, some [34,35,37,39,41,46] used the IOTF criteria, others [40,44,47] used the WHO one, and other [45] used the CDC criteria.

When using logistic regression, in most of the cases, [34,35,37,41,42,45,46] a stepwise variable selection is performed from a pull of predictor variables to select the final ones to use in the definitive model or models. In one case [39,44], a score is derived “by hand” by combining odds-ratios obtained from simple logistic regressions of different variables and then used in a simple [39] or multiple [44] logistic regression to estimate its odds-ratio. In two other cases [36,43], the predictor variables are predefined, and in one case [40] several predefined predictor variables are used at the beginning, but then, the model is rederived with only the significant ones.

As regarding the predictor variables, the most popular ones, in decreasing order, are parental BMI (8 times), sex and birth weight (7 times), smoking mother during gestation (6 times), weight gain at some previous period (5 times), parental education (4 times), exclusive breastfeeding during some initial period (3 times), etc. Sometimes, versions of these variables are used, like categorized ones (e.g., obesity instead of BMI) or standardized ones. Some other times, mother’s version (instead of parental ones) are used, e.g., mother’s BMI, or mother’s education. There are two cases where a set of genetic polymorphisms are used; in one case [37], incorporated as a score obtained as sum of risk alleles, they appeared to add no significant predictive capacity, but in the other, [47] in the form of components of a Multiple Component Analysis (MCA), they did.

The cohorts used in the derivation of the models are of variable origin: Netherlands [34], UK [36,41,42], Finland [37,43], Germany [38,40], Greece [39,44], USA (Latino community) [45], and Spain. [47] In the case of the metanalysis previously mentioned [35], the 10 different cohorts are also from multiple countries: UK, France, Finland, Sweden, USA, and Seychelles. We can see that most of the work has been performed in developed countries with mostly Caucasian samples, which limits their applicability. The sizes of the cohorts are also variable: They range from 166 [45] to around 13,000. [41] The metanalysis [35] includes more than 47,000 cases in the 10 cohorts.

In terms of model validation, some of the models [35,37,41,43,44,46] were externally validated (as a matter of fact, the works by Manios et al. [44] and Redsell et al. [46] are external validations of the previous models described in Manios et al. [39] and Weng et al. [41]), while other models were internally validated through bootstrap [34,45,47] or cross-validation [38,40]. In two cases, [42,43] both internal and external validation was used, while in one case [36] no validation was performed at all.

If we focus in the comparison of performances of the different logistic regression models, we can use the AUCROC (that equals the so-called c-index or concordance index) as a criterion for discrimination. Depending on where the linear predictor threshold of the model is set to assign one category or its alternative to the predictions, we can have very different sensitivities and specificities, as well as PPV (precision) and NPVs; to select the threshold we must take into account the purpose of the model, as well as the possible costs of false positives and/or false negatives. However, as a *global* measure of the discriminative capacity of the model, before its practical application by selecting a threshold, the AUCROC is a well-established criterion. Obviously, for two models with the same AUROC, one internally validated and the other externally validated, we will prefer the one externally validated, especially if it is with a large, unrelated cohort, because it will approximate more closely a real-life prediction than the internal validation that is based on data reutilization.

In this way, the models of the different works using external validation would be ranked in the following order of decreasing AUROC: Santorelli et al. [42] (0.89), Morandi et al. [37] (0.79), Druet et al. [35] (0.77), Weng et al. [41] (0.75), Redsell et al. [46] (0.67), and Manios et al. [44] (0.64). In the case of the paper by Graversen et al. [43], the AUROC is provided only for the internal validation. These values should be taken with caution, given that they do not compare the same “difficulty” in prediction, e.g., if the testing cohort is very similar to the training one, a very large AUROC could be obtained very easily; for example, an external validation with a different cohort to the training one is a more demanding task that an external validation with a random split of the same cohort, even if the latter is not used for training. Moreover, the difficulty depends on the relatedness between the predictor and target variables, e.g., the prediction of obesity at age 9 is more difficult if the predictor variable is weight gain between 0 and 1 years than if the predictor variable is weight gain between 7 and 8 years.

On the other hand, the ranking of models for internal validation by decreasing AUROC is Robson et al. [45] (0.78) and Steur et al. [34] (0.75). Mayr et al. [38] and Pei et al. [40] do not provide AUROC values. In principle, the evidence of predictive capacity of these models is weaker given that they have not been externally validated.

Finally, we should mention that, in terms of the type of prediction, all the models have a longitudinal setting, that is, they aim at predicting the endpoint *in the future* from *predictor variables taken in a previous point in time*, at least partially, e.g., predict overweight at 8 years using birth weight and mother smoking at gestation. These are designated in Table 1 as “L” type of prediction. The only exception is the work by Cortés-Marín et al., which has no predictive but explanatory purpose and therefore uses a cross-sectional setting, where the predictor variables are taken at the same time than the endpoint. This is designated in Table 1 as “CS” type of prediction. Here, the aim is to obtain the relative strengths of associations of variables of different domains with putative explanatory character (diet, age, sex, genetic polymorphisms, microbiota), although given the cross-sectional setting of the model, no demonstration of causality can be obtained from it but rather of putative variables to consider for a further test with a longitudinal setting.

## 5. Machine Learning Models to Predict Childhood/Adolescent Obesity Based on BMI

In this section, we will review the ML models derived to predict BMI (regression) and/or categorized versions of it (classification), e.g., normal-weight, overweight, obesity, etc.

To our knowledge, there are only two previous reviews of ML models to predict childhood/adolescent obesity. One early paper in 2010 by Adnan et al. [54] described the scarce work performed before it; another very recent paper [55] reviews the work up to 2020, together with the area of computerized decision support for the prevention and treatment of childhood obesity. However, the latter paper, being arranged as a systematic review, lacks many of the publications in the area of ML, and some of the ones described there could be more appropriately defined as statistical models (e.g., generalized linear mixed models and linear and logistic regression) or are targeted to the prediction of physical activity in children.

In what follows, we will use more or less a chronological order in the description of the works conducted in the area. As we will see, the field has experienced an explosion very recently, especially through the use of electronic health records (EHR) as sources of very large datasets. ML methods will be abbreviated as in Section 2. Table 2 summarizes the main features of the models that will be described.

The first attempts to use ML to predict childhood obesity are those of Novak and Bigec, back in 1995 [56] and 1996 [57]. In these papers, they describe the use of ANN to predict childhood obesity. However, the work is of preliminary nature and is more a description of the ANN theory and method, without providing a description of the results of a particular model derived from a particular sample.

This work was followed by that of Zhang et al. in 2009 [58]. Here, the aim is to compare the performance of ML models with the traditional logistic regression model. By using an UK cohort (the so-called Wirral database of >16,000 children), they developed several models to predict overweight at 3 years from previous data, using predictor variables available at 8 months or at 2 years. These variables were all child features like sex, BMI at 8 months, adjusted SDS of height at different visits, weight gain between pairs of visits, etc. Different ML methods were used: DT, Association Rules, ANN, Linear SVM, RFB (Radial Basis Function) SVM, BN, and NB. In the case of the prediction at 8 months, the ANN showed the largest accuracy, although the RBF (Radial Basis Function) SVM displayed the largest sensitivity (probably more useful for clinical purposes). For the prediction at 2 years, the largest accuracy was obtained with the Bayesian methods, although the largest sensitivity was observed in the case of RBF SVM again. Logistic regression had the largest specificity, but the sensitivity and accuracy were much worse than the ML models. They also derived models to predict obesity, but the quality of them was very low. No validation was performed in any of the models developed.

A work in 2011 by Rehkopf et al. [59] used and American cohort (the NHLBI Growth and Health Study) of ca. 2000 white or black girls 8 or 9 years old that were followed for 10 years to predict the change from 9 to 19 years in the CDC BMI percentile and the transition from normal-weight to overweight or obese by means of RF models. They took 41 predictor variables from different domains: diet, physical activity, psychological, and social and parent health in order. They applied variable importance techniques by permutation to estimate the relative importance of these variables. For the first outcome, body dissatisfaction, drive for thinness, physical appearance (psychological), income and parental education (social), and other psychological variables were the most important variables. In the case of the transition to overweight or obesity, the most important predictor was income, followed by psychological variables. Again, no internal/external validation of the model was performed.

Following their review in 2010 [54], Adnan et al. published in 2012 three papers in this area [60,61,62] to predict the nutritional status (normal-weight, overweight and obese) by means of NB and a cohort of 140 Malaysian children 9–11 years old. They applied 19 predictor variables of different domains obtained from literature review: children features, lifestyle (including physical activity and diet), and family/environment. In the first work [60], they observed that the use of these variables improved the accuracy of obesity prediction by NB as compared to the work by Zhang et al. [58]. This approach was improved in the second paper [61] by using a genetic algorithm to select predictor variables in order to avoid the problem in NB with many variables where the predicted posterior probabilities turn to zero each time at least one of the predictor variables prior probability is zero. The third paper [62] adopted two additional methods for variable selection for NB models: variable importance with CART and Euclidean distances. The models were not validated in any of the papers.

Another work from 2012 is that of Lazarou et al., [63] where diet variables were used to predict overweight + obesity vs. normal-weight. A Cypriot cohort of ca. 600 children 10–12 years old was used with a cross-sectional setting. They used questionnaires of eating frequencies of food groups as predictor variables (fried food, fish and seafood, delicatessen meat, soft drinks, and sweets and junk food). By developing many DTs, for both boys and girls, they were able to derive rules of overweight + obesity risk as a function of diet patterns and sex. The approach was validated by bootstrap, but the results were not shown. Finally, they developed logistic regression models using as predictor variables PCA components of the diet variables; only one of the PC of the girls model was significant.

One paper in 2014 by Pochini et al. [64] predicted overweight and obesity in high-school students (14–18 years old) from 9 lifestyle predictor variables, using both logistic regression and DT, again, in a cross-sectional setting. The sample modeled was a cohort of ca. 15,000 high-school students in Columbia, USA (from the 2011 CDC Youth Behavior Risk Survey). For obesity, logistic regression significant factors were consumption of fruit/vegetables, smoking, being physically active, having regular breakfast, drinking fruit juice, and drinking soda; the remaining variables in the DT after pruning were physically active and tobacco. For the overweight prediction, the logistic significant variables were having regular breakfast and being physically active. For the DT, no variable remained after pruning; before pruning, the variables were breakfast, fruit juice, and sleep. In the case of the DT, the models were externally validated with a 30% of the original sample.

Dugan et al. [65], in 2015, used multiple ML methods to predict obesity at 2 years using predictor variables obtained before that age. The data came from a clinical decision support system, CHICA, that contained information from a multiethnic cohort in USA of >7000 children. Random Tree, RF, J48, ID3, NB, and BN were tried out. The best performing algorithm was ID3, with an accuracy of 85%, sensitivity of 89%, PPV of 84%, and NPV of 88%. Using some sort of variable importance by removing variable by variable, they found that the strongest predictors were overweight before 24 months, followed by being very tall before 6 months. All the models were internally validated through 10-fold cross-validation.

In 2016, a paper by Lingren et al. [66] was published aimed at the identification of putative cases of severe early childhood obesity from children 1–6 years old above the 99th BMI percentile, to separate them from those due to medications, pathologies, etc. The objective was to develop a cohort for further genotyping studies, in order to understand the genetic basis for severe early childhood obesity. Therefore, they attempted to optimize the PPV, in order to be most effective in the detection of these children. The dataset used corresponded to a cohort of >5000 of EHR from two children hospitals, one in Boston and another in Cincinnati. The predictor variables used were structured data (demographics, anthropometrics, ICD-9 diagnosis codes, and medications) as well as unstructured data (narrative) by NLP. They used both rule-based methods and ML methods (SVM and NB) that were tested in an external split of the original data. In general, the rule-based method worked better, but the ML one had more flexibility to leverage PPV and sensibility and to select variable sets.

Abdullah et al. published a paper [67] in 2017, where they used ML to predict obesity at 12 years from a Malaysian cohort of >4000 children 12 years old. The predictor variables were obtained from questionnaires and included three domains: socio-demographic, physical activity, and diet. Multiple methods for variable selection were tested, as well as multiple ML methods: BN, DT (J48), NB, ANN, and SVM. The best results were obtained with J48, together with consistency + linear forward variable selection. In this case, the models were not validated.

A later paper in the same year by Rios-Julián et al. [68] attempted to predict obesity + overweight (following the CDC criteria) vs. normal-weight by using BMI and other anthropometric variables in a community of Me’Phaa ethnicity in Mexico. They modeled a cohort of 221 children 6–13 years old by using different ML models: J48, logistic model trees, ANN, RF, and logistic regression. Three groups of variables were tried on: all; all but skinfold thickness; and sex, age, height, weight, BMI, and skinfold thickness. They obtained not very different results for the different variable groups and models, and in general, all the models yielded excellent predictions. All the models were internally validated by 10-fold cross-validation.

Moreover, in 2017 Wiechman et al. published a paper [69] that used DTs (C4.5 type) to gain insight on the factors influencing child obesity in Hispanic preschoolers in the USA. The sample analyzed was a cohort of children of 238 families, 2–5 years old, of Hispanic ethnicity. They develop shallow C4.5 decision trees to predict overweight by using variables from different domains: demographics, caregiver feeding style, feeding practices, home environment, dietary information, beverage consumption, social support, family life, integrated behavior model, and spousal support. They found some clues for obesity development: If the mother cares for the child or if she works but the father has high-level education, the child has less probability of being overweight. If the child is fed to avoid tantrums, the child tends to be more obese. The models were not validated.

The last paper in 2017 is that of Zheng and Ruggiero [70]. They used a dataset comprising a cohort of >5000 high-school (14–18 years) students in the USA. They predicted obesity between 14 and 18 years from 9 variables within three different domains: energy update, physical activity, and sedentary behavior. They used logistic regression, DT, kNN, and ANN. The best models were ANN and kNN, and all the ML models performed much better than logistic regression. All were internally validated by 10-fold cross-validation.

The year 2019 saw an explosion of ML and DL models to predict childhood obesity. We have identified up to 7 papers in this area, together with other aimed at related endpoints that will be described in the next section. Several of them make use of EHR as sources of data. We finish this section by describing these works.

An example of DL models is that of Gupta et al. [71] They used a cohort of EHR from ca. 68,000 children/adolescents with visits to medical centers for at least 5 years, in order to predict BMI and obesity from 3 to 20 years in groups of 3 consecutive years using data from the 3 previous years, resulting in multiple models. Recurrent NN of the LSTM type were used, with predictor variables from the EHR including medical conditions observed, drugs prescribed, procedures requested, and measurements taken, together with static demographic data. Data was split into three subsets: 60% for training, 20% for hyperparameter validation, and 20% for external validation. The whole training dataset was used to train a global model, and then, by transfer learning, specialized models for each sub-cohort were obtained by retraining the global model with the corresponding subset of data. In order to identify important variables, they used embedding, while to identify important time intervals, they used attention techniques. The RNN was compared with RF and linear regression, which do not take into account the longitudinal information, and the RNN gave a much better performance. The performance of the models decays with the temporal distance between the acquisition of the predictor variables and the time of BMI prediction in the future, as expected.

Another work that used EHR data is that of Hammond et al. [72], who used a multiethnic cohort obtained from multiple providers in a safety net in New York city that included >3000 children. The authors predict obesity at year 5 by using logistic penalized regression, RF, and GB. In addition, obesity was predicted by deriving regression models for z-BMI using LASSO, RF, and GB and applying an obesity cutoff for the z-BMI predicted. They used feature engineering to generate predictor variables from the EHR: demographic information, home address, vital signs, and medications from the children when they were < 2 years old and from the mother vital signs, diagnosis codes; procedures; and laboratory results before, during, and post-pregnancy. They developed different models for boys and girls. The most important predictors were weight-for-length z-score, BMI between 19 and 24 months, and the last BMI measure before age 2. The best models have an AUCROC of 81.7% for girls and 76.1% for boys. Internal validation was conducted by bootstrap CV and external validation with a previously selected test split.

One case of work aiming at understanding risk factors for childhood obesity is that of Lee et al. [73]. They used a South Korean longitudinal cohort of ca. 1 million children and used DT models to predict obesity vs. normal-weight between 24 and 80 months (overweight children are removed). They used a set of 21 predictor variables of different domains: socioeconomic status (SES (modelled after attending medical aid or not), maternal factors (e.g., pregestational obesity, abdominal obesity, hypertension, smoking, etc.), paternal factors (obesity, abdominal obesity, and hypertension), and child factors (preterm, exclusive breastfeeding, high consumption of sugar sweetened beverages, etc.)) The model was externally validated with a 40% test split, resulting in an accuracy of 93%. By using a CHAID-type of variable selection, the most important predictor variable was mother obesity, followed by parental obesity and SES; other important factors were old pregnancy and gestational diabetes and hypertension. Child factors were exclusive breastfeeding, consumption of sugar-sweetened beverages, and irregular breakfasting. Interestingly, they observed that child’s z-score for weight at birth and z-score for weight-for-height were not selected.

A South-Korean dataset was also used in order to understand factors affecting obesity is that of Kim et al. [76], although in this case it deals with *adolescent* obesity. They used a cohort of >11,000 students from South Korea and 19 predictor variables from questionnaires of different domains: sociological, anthropometric, smartphone use, obesity, other. They predict the three categories of BMI: underweight, normal, and overweight, by means of a General Bayesian Network (GBN), and compare it with many different ML methods resulting in GBN displaying the best fit: the best accuracy is 53.7%, and the AUCROC is 0.758. No validation is performed. The variable most related to BMI class is pocket money. More interestingly, they use the GBN to perform a “what-if” analysis by modifying the values of different variables or combination of variables in order to get an understanding of putative mechanisms for risk of obesity. For instance, the combination of high pocket money and low wealth increases a lot the probability of obesity, etc.

An adolescent cohort was also used by Singh et al. [75] but in this case from the UK. The Millenium cohort of UK of children born between 2000 and 2001, particularly the subsets MC2 to MC5, was modeled in order to predict the BMI at 14 years (MC6). The data was externally validated with a test split of 25%. Linear SVM, linear regression, and ANN were tried, and the best performance was obtained by the ANN, followed by the SVM.

A work that uses XGBoost is that of Pang et al. [77]. The authors predict obesity in the period 2–7 years from data in windows in the 0–2 years period with a cohort of ca. 27,000 children from Philadelphia in the Pediatric Big Data repository. Variables included vital signs, laboratory values, and provider information, resulting in a total of 102 predictors. Data was divided into train 1 (40%), train 2 (40%), and hold-out (20%) to determine hyperparameters iteratively and train/test the model. Different ML models were tried, and the best was XGBoost, giving an AUCROC of 0.81, and for the threshold that gives a recall of 0.8, the precision, F1, accuracy, and specificity were 30.9%, 44.6%, 66.14%, and 63.27%, respectively. They analyzed the models with variable importance techniques, resulting in weight-for-height at month 24, weight at month 24, weight for height at month 18, and race being the most important ones. Different races, ethnicities, and caregivers had different importance distributions. Using sensitivity analysis, it was observed that the prediction of obesity at later times degrades, as expected.

An interesting alternative set of predictive variables to the ones described so far is the use of neuroimaging biomarkers. This is the case of the work by Park et al., [74] who used resting-state functional magnetic resonance imaging (rs-fMRI) to derive predictive models for BMI progression (and indirectly future BMI) of adolescents. A cohort of 76 individuals from the Enhanced Nathan Kline Institute Rockland Sample (NKI-RS) database of white and African American preadolescents (average age of 11.94 years) was used. Their BMI was measured in a first visit, followed by a second visit about 1.5 years later. From the fMRI of their brain in the first visit, both considering subcortical volume and cortical surface, 379 Degree-Centrality (DC) values of different parts of the brain were extracted. These were used with LASSO to predict the BMI progression (DeltaBMI/Deltat) and indirectly BMI in the second visit. Only six DC remained after the variable selection in the LASSO. These variables were entered in a linear regression model. The model was internally validated with leave-one CV, giving and Intra Class Correlation (ICC) for DeltaBMI of 0.70, and ICC for BMI of 0.98, and (when predicting the binary variable increase/decrease of BMI) an AUCROC of 0.82. Brain regions of the selected DCs were correlated with the eating disorder, anxiety, and depression. The approach was applied to a local South Korean dataset of 22 young adults (average age of 21.4), and the results were similar, suggesting robustness of the first model.

## 6. Machine Learning Models to Predict Related Outcomes

Some other works in the literature make use of ML to derive predictive models not of BMI or BMI categories, but of related endpoints. Table 3 summarizes these models.

For instance, a work by Duran et al. [82] describes the use of ANN models to predict body fat percentage (BF%) and its excess (BF% above 85th percentile), which is an alternative measure of obesity to those based on BMI. A cohort ca. 2000 non-Hispanic white children less than 20 years old were used here. Different models were derived for boys and girls. The predictors used were age, height, weight, and waist circumference. The ANN were compared with the prediction using z-BMI and z-WC. In the case of boys, ANN has better accuracy, sensibility, and specificity than the simple models, especially the z-WC one; in the case of girls, the ANN performs similarly to the z-BMI one and better than the z-WC one. The models were internally validated and externally validated with a test split. 

On the other hand, there are models aimed at the prediction of the success of therapies or treatments to decrease childhood obesity. One case is described in a work by Hasan et al. [79], where they used RNN (both LSTM and GRU types) and probabilistic models to try to predict the positive or negative reception by obese adolescents of communication sequences by a counselor in interviews to promote weight reduction behavior. The authors used a dataset of 129 motivational interviews between a counselor and an adolescent (accompanied by a caregiver) for promoting weight reduction behavior. These interviews included 50,239 encoded sequences of utterances ending or not in a positive change talk or positive commitment language by the adolescent or caregiver. Given the high imbalance of the sequences of utterances (most of them are successful ones), they evaluated the models through either synthetic oversampling of the negative sequences or under-sampling of the positive ones. The models were trained with 80% of the data and externally evaluated with 20% of the data. In the case of under sampling, the LSTM models with target replication (LSTM-TR) resulted in the best models in terms of F1, precision, and recall. The probabilistic models were much worse. When using oversampling, the LSTM-TR was again the best model. These models can therefore be used to design communication strategies that achieve the best success.

Another example of prediction of therapy success is the work by Öksüz et al. [80] They used a cohort of 20 overweight or obese children 11–16 years old in Switzerland to predict the success of a weight-decrease 6-months therapy (defined as BMI after therapy < 0.4 BMI units than before). As predictors, they measured the heart rate at several intervals during a run test and a cooldown period, plus weight, age, BMI, and height. They tried different ML methods: several SVM, kNN, DT, and GB. Nested cross validation was used to train and internally validate the models given the small sample size. The best model used linear SVM, giving an accuracy of 85%. They used permutation tests to estimate the relative importance of the predictors, and several heart rate ones are the most important. These ML models performed better than the prediction of two domain experts.

A related task is the detection from EHR of attention by pediatricians to childhood obesity and associated medical risks. This is the case of the paper by Turer et al. [81]. They used a dataset of doctor visits of >7000 overweight/obese children 6–12 years old in several centers in Texas. They developed a rule-based classification algorithm to detect from EHR doctor’s behaviors that indicate therapeutic “attention towards excess BMI”, “attention towards excess BMI + comorbidities (medical risk)”, and “no attention”. They used different types of evidence, in addition to pathology codes, from EHR indices: diagnosis codes, orders for laboratories, medications, and referrals. The algorithm was externally validated by manual review of EHR data of 309 additional visits. Sensitivity to BMI alone was 96%, while to BMI/Medical risk was 96.1%.

We end this section with an interesting paper by Nau et al. [78] describing a predictive model for obesogenic vs. obesoprotective community environments. Here, the aim is predicting not the obesity for a particular child or adolescent, but rather if the features of a community are those that foster childhood obesity within it, or on the contrary, they protect against it. These authors analyzed 99 communities in Pennsylvania, 50 of them in the high quartile of child obesity prevalence and 49 others other in the lowest quartile. Therefore, it uses community-aggregated data to try to predict obesogenic vs. obesoprotective communities. They used 44 variables as potential predictors in different domains: food services, social, physical activity establishments, and land use. They used variable importance measures with RF to identify the most important variables. A total of 13 were deemed important above noise; unemployment was the most important, followed by population density, social disorganization, proportion of people with less than high school education, population change, no car ownership, etc. These are physical activity and social variables. The most important food services variables are counts of snacks stores and counts of fast food chains score. Models were also obtained without social variables that are considered causal of the others; the results gave similar ranking of the other variables. It seems that well-off communities are more protected against obesity. It was also observed that classification accuracies were different for high and low obesity communities, indicating different structures/hierarchy of variables for these two groups. The models, however, were not internally or externally validated.

## 7. Discussion

In the present Review, we have seen a large amount of models to predict childhood/adolescent obesity. We have grouped them into two types: statistical ones and ML ones. The former models use traditional statistical techniques, mainly logistic regression, [34,35,36,37,39,40,41,42,43,44,45,46] although there are cases using linear regression [40], quantile regression [38], and ordinal logistic regression. [47] The ML models use a wide variety of ML methods: ANN [56,57,58,67,68,70,75], SVM [58,66,67], DT [58,64,65,67,68,69,70,73], NB [58,60,61,62,66,67], BN [58,65,67,76], LASSO [72,74], kNN [70], RF [59,65,68,72], GBM [72,77], and DL (RNN [71]).

In general, when in the same work logistic/linear regression is compared with ML models when fitting the same dataset [58,64,68,70,72,75], the latter give better results than the former in terms of prediction performance. This confirms that ML techniques are able to yield better predictions, not just by fitting better the training set but also through giving better results in internal and/or external validations.

On the other hand, if we analyze the models in terms of predictor variables, we see that the statistical models make use in most of the cases of a reduced set of well-established risk factors for childhood obesity: parental BMI, sex and birth weight, smoking mother during gestation, weight gain at some previous period, parental education, exclusive breastfeeding during some initial period, etc. Only the work by Cortés-Martín et al. [47] uses a wider set of predictor variables, including a Mediterranean diet score, multiple SNPs, and a marker of microbiota (urolithin metabotype), in addition to sex, age, and ethnicity. On the contrary, in multiple ML models, we observe other types of variables, alone or in combination with the “traditional” predictor variables. For example, the work of Rehkopf et al. [59] uses psychological predictor variables, that of Lazarou et al. [63] focuses mainly on diet, while that of Park et al. [74] utilizes rs-fMRI predictor variables. There are also several papers that use lifestyle variables (including both diet- and physical activity-related variables) [59,60,61,62,64,69,70]. Works that stand out for their use of specially wide sets of multidomain predictor variables are those of Rehkopf et al. [59] (diet; physical activity; and psychological, social, and parental health); Wiechman et al. [69] (demographics, caregiver feeding style, feeding practices, home environment, diet, social support, spousal support, family life, etc.); and Kim et al. [76] (wealth, smartphone use, pocket money, academic performance, sleeping quality, etc.) The latter work is interesting also because it makes a “what-if” analysis where some variables are modified, and their concerted effect on the predicted obesity is evaluated; this is an interesting approach to use ML models as simulation tools to suggest possible therapeutic or preventive interventions.

Therefore, we could say that the statistical models are probably more oriented towards earlier ages, where the number of factors affecting is less variable, or to predicting shorter times in the future. We would be mainly doing a short extrapolation of the BMI curve: Obese children would be those who were obese some short time before, and in the case of babies or early age children, gestational factors like smoking mother or gestational diabetes would also be of importance. These are simpler models with immediate implementation in the clinics, as they contain a small number of easily retrieved predictor variables. On the contrary, once the multidomain factors of obesity, like diet, physical activity, psychological variables, genetic, family environment, sociological, etc., enter the scene, which takes place in late childhood or adolescence, ML models are more appropriate. This is also for predictions spanning large periods of time, like the model by Gupta et al. that was developed to predict BMI and obesity from 3 to 20 years, or when we require higher accuracy in the prediction. In addition to prediction purposes, these ML models are useful in that they can be used to rank these wide sets of variables by importance, thus allowing to better identify the strongest risk factors and generate new ideas for future preventive interventions [59,71,73,74,77,83]. In the case of longitudinal models, the strongest influence times can also be derived through attention techniques [71].

A specially interesting situation from the point of view of predictor variables are the ML models that use EHR [65,66,71,72,77], since they appear as very powerful approaches to predict childhood/adolescent obesity by tapping from the large databases of medical records with many patients and extended sets of predictor variables, including measurements, drug prescriptions, conditions observed, and procedures requested. These are especially amenable of DL techniques of the RNN type, which are specialized in dealing with time serial data like this. As described above, one case in the models presented here is that of Gupta et al. [71], where they were able to deliver excellent predictions of BMI and obesity along the whole childhood and adolescence growth curve. Another interesting use of RNN in this framework would be the extraction of information from narrative data in the medical records by means of NLP; an example of predictor variables extracted through NLP is the work by Lingren et al. [66].

These ML/DL models using EHR could be implemented in hospitals and primary health care centers to provide predictions and alerts through *dynamic*, *online* training. By this, we mean a model that is fed continuously with new data and is retrained periodically to enhance its predictions with the new data. This is opposed to *static*, *offline* training where the model is fit with a definite dataset and only once and forever. All the models we have reviewed are in the last category.

EHR offer also very interesting opportunities as Big Data sources for remining through ML/DL models. For example, we have seen the case of the work by Lingren et al. [66] where the EHR was exploited to identify a cohort of severe early childhood obesity for further genotyping efforts. Many other applications are possible, like analyzing and predicting comorbidities through statistical network analysis [84,85,86], phenotyping, diagnosing, pharmacoepidemiology and pharmacovigilance, etc. [87].

In addition, we should mention the fruitful application of ML/DL models to the field of childhood obesity prevention, not just through the identification of risk subpopulations but through the analysis of different aspects of the preventive intervention. We have seen that these models can be useful to optimize obesity prevention strategies [79], predict its success [80], and identify doctor’s behaviors attentive or not of childhood obesity and related risks in the clinics [81] and, from a community point of view, social environments with obesogenic properties that should be targeted with preventive governmental policies [78].

To summarize, ML/DL approaches offer extraordinary advantages and new insights for childhood and adolescent obesity prediction and prevention over statistical methods. The following points summarize them:

- They have increased the prediction accuracy over the statistical models, given their ability to model complex, nonlinear relationships between variables, as well as the very large number of parameters that they contain, especially in the case of DL, avoiding the saturation in prediction performance. This is always beneficial irrespective of the application, as the more accurate is a model the more practical it is in real life.
- They allow to model directly and automatically high-dimensional data, which is not possible in the case of traditional statistical models. For the latter, one has to make use of questionable variable selection techniques, and at the cost of making biased inferences.
- They have expanded the predictor variable set from the “traditional” one in statistical models (well-established risk factors) to a much wider, multidomain one: psychological, diet, social, lifestyle, smartphone use, academic performance, sleeping quality, drug prescriptions, spousal support, rs-fMRI, and other domains of variables. In this way, they have been able to find new insights about novel “risk” factors of a completely new nature.
- They are able to use new complex data sources other than numeric ones: text, images, RMN, social media, etc.; this is especially the case with DL, which can use that data directly, without previous “feature engineering”. This is another way to expand the number and domain of predictor variables.
- They (especially DL) are appropriate tools for the modeling of EHR, as described in the examples reviewed. In particular, the EHR offer an incredible opportunity for remining efforts, in order to find new insights from these data sources that can suggest opportunities for further research and therapeutic approaches.
- They have provided new applications, besides the prediction of risk subpopulations, e.g., identification of samples for genotyping from EHR, optimization of utterances in counseling interventions to decrease adolescent obesity, analysis of pediatrician attention to obesity and related risks from EHR, etc.
- They have been used not just as prediction or explanatory tools but also as simulation tools, so that they can be used to get new insights about possible therapeutic approaches.

To be fully fair, we should as well mention the *disadvantages* of ML methods over statistical ones. The first one is that making statistical inferences (parameter estimation and hypothesis test) in these models is more complicated than in statistical models. However, it is not impossible, and resampling and simulation techniques could be used if required. Another drawback of ML models is that they are more difficult to interpret, and they are typically called “black-box” type of models. This is an area of intense research, and we have seen above several examples of techniques to solve this problem, namely, the techniques of variable importance, embedding, and attention.

Seeing the advantages and disadvantages of both types of methods, we can ask: when is ML more appropriate, and when are statistical models? The following patterns have emerged:

- If the interest is mainly in interpretability, inference, and simple models of reduced numbers of predictor variables, instead of predictive performance, statistical models should be more appropriate. Again, this is more the case with early childhood and for clinical applications not requiring high accuracy.
- The opposite is applicable: If we want to have very good predictive performance and are less worried about interpretability and inference, ML should be used.
- If we have a high-dimensional sample, ML should be used, for example, if we want to analyze a wide, multidomain set of predictor variables. This is more the case with exploratory studies from which we want to gain new insights about new risk factors: psychological, social, genetics or genomics, microbiome and metagenomics, neuroimaging, diet, lifestyle, etc. As said above, these models are more likely to be relevant as the child grows and especially during adolescence.
- If we want to use complex data as predictors (images, text, time series, social media, etc.) DL is the one to go for.
- If we want to use EHR, ML (DL) should be applied.
- It is likely that new applications will go mainly through the use of ML or DL, as these are more powerful to tap from Big Data sources: Internet, social media, mobile and wireless devices, sensors, etc. These applications would include computerized decision support systems, simulation applications, novel preventive interventions, analysis of social obesogenic environments, etc.

Hopefully this Review will help the wide set of researchers in the field, including pediatricians, nurses, nutritionists, statisticians, data scientists, engineers, and epidemiologists, to get an updated view of these novel approaches and the opportunities they open, in order to approach in a more effective and creative way the prevention of childhood and adolescent obesity. We are at the beginning of a qualitatively new phase that can revolutionize this field in the near future.

## Figures and Tables

**Table 1:** Summary of statistical models.

Article (year)	Training Set Size	Number of Predictors	Country	Outcome Predicted *	Statistical Method	Validation	Type of Prediction **
Steur et al. [34] (2011)	1687	6	The Netherlands	OW 8 years	Stepwise Logistic Regression	Bootstrap	L
Druet et al. [35] (2012)	47,661; 8236	4	UK, France, Finland, Sweden, US, Seychelles	Childhood OB OR; Childhood OB	Metanalysis; Stepwise Logistic Regression	External	L
Levine et al. [36] (2012)	Not specified	5	UK	OB 5 years	Logistic regression	None	L
Morandi et al. [37] (2012)	4032	6	Finland	OB 7 years; OW 7 years; OB 16 years; OW 16 years; OB 7 and 16 years; OW 7 and 16 years	Stepwise Logistic Regression	External	L
Mayr et al. [38] (2012)	Not specified	10	Germany	Childhood OB prediction intervals	Quantile regression with boosting	Simulation and Internal	L
Manios et al. [39] (2013)	2294	5	Greece	OB 9–13 years	Logistic regression	None	L
Pei et al. [40] (2013)	1515	5	Germany	zBMI 10 years; OW 10 years	Linear regression; Logistic regression	Cross-validation	L
Weng et al. [41] (2013)	10,810	7	UK	OW 3 years	Stepwise Logistic Regression	External	L
Santorelli et al. [42] (2013)	1735	4	UK	OB 2 years	Stepwise Logistic Regression	External	L
Graversen et al. [43] (2015)	4111	3	Finland	OW adolescence	Logistic Regression	Bootstrap, External	L
Manios et al. [44] (2016)	5946	5	Greece	OB 6–15 years	Logistic Regression	None	L
Robson et al. [45] (2016)	166	5	US	OB 5 years	Stepwise Logistic Regression	Bootstrap	L
Redsell et al. [46] (2016)	980	7	UK	OW 5 years	Logistic regression	External validation of Weng et al. [41]	L
Cortés-Martín et al. [47] (2020)	415	7	Spain	NW/OW/OB 5–17 years	Ordinal Logistic Regression	Bootstrap	CS

* NW = normal weight; OW = overweight; OB = obese; ** L = longitudinal model; CS = cross-sectional model.

**Table 2:** Machine Learning (ML) models to predict BMI or its categories.

Article (year)	Training Set Size	Number of Predictors *	Country	Outcome Predicted **	ML Method	Validation	Type of Prediction ***
Novak and Bigec [56] (1995)	ND ****	ND	Slovenia	Childhood OB	ANN	Not described	ND
Novak and Bigec [57] (1996)	ND	ND	Slovenia	Childhood OB	ANN	Not described	ND
Zhang et al. [58] (2009)	16,523	10	UK	OW 3 years	DT, ANN, NB, BN, SVM, association rules, logistic regression	None	L
Rehkopf et al. [59] (2011)	2150	41	US	Girls Change in BMI percentile (9 to 19 years); onset of OW or OB	RF	None	L
Adnan et al. [60] (2012)	140	20	Malaysia	Children OB 9–11 years	NB	None	CS
Adnan et al. [61] (2012)	320	8	Malaysia	Children OW/OB	NB	None	CS
Adnan et al. [62] (2012)	180	19	Malaysia	Children OW/OB	NB	None	CS
Lazarou et al. [63] (2012)	600	5	Cyprus	OW 10–12 years	DT	Bootstrap	CS
Pochini et al. [64] (2014)	15,425	9	US	OW 14–18 years; OB 14–18 years	DT; Logistic Regression	External	CS
Dugan et al. [65] (2015)	7519	167	US	OB 2 years	DT; RF; NB; BN	Cross-validation	L
Lingren et al. [66] (2016)	5857	9	US	Severe OB 1–6 years	Rule based; SVM; NB	External	CS
Abdullah et al. [67] (2017)	4245	29	Malaysia	OB 12 years	BN; DT; NB; ANN; SVM	None	L
Rios-Julian et al. [68] (2017)	221	16	Mexico	OW 6–13 years	DT; Logistic Model Trees; ANN; RF; Logistic Regression	None	CS
Wiechmann et al. [69] (2017)	238	ND	US	OW 2–5 years	DT	None	CS
Zheng and Rugggiero [70] (2017)	5127	9	US	OB 14–18 years	Logistic Regression; DT; kNN; ANN	Cross-validation	CS
Gupta et al. [71] (2019)	40,817	1737	US	BMI and OB 3–20 years	DL(RNN)	External	L
Hammond et al. [72] (2019)	2759	19,290		OB 5 years	Logistic penalized Regression; RF; GBM; LASSO	Bootstrap cross-validation; External	L
Lee et al. [73] (2019)	~ 600,000	21	South Korea	OB 24–80 months	DT	External	L
Park et al. [74] (2019)	76	379	US	BMI progression in childhood	LASSO	Cross-validation; External	L
Singh and Tawfit et al. [75] (2019)	ND	ND	UK	BMI 14 years	Linear Regression; ANN	External	L
Kim et al. [76] (2019)	11,206	19	South Korea	BMI categories	BN	None	CS
Pang et al. [77] (2019)	10,881	102	US	OB 2–7 years	GBM	External	L

* When several models are derived, the largest number of predictors is reported; ** NW = normal weight; OW = overweight; OB = obese; *** L = longitudinal model; CS = cross-sectional model; **** ND = Not described. ML Method abbreviation as in Section 2.

**Table 3:** Summary of ML models to predict BMI-related outcomes.

Article (Year)	Training Set Size	Country	Outcome Predicted	ML Method	Validation
Nau et al. [78] (2015)	99 (communities)	US	Obesogenic environment	RF	None
Hasan et al. [79] (2018)	40191 (utterances)	US	Success of communication strategies to promote weight reduction behavior	DL	External
Öksüz et al. [80] (2018)	20	Switzerland	Success of weight decrease therapy	SVM, kNN, DT, GBM	Cross-validation
Turer et al. [81] (2018)	7192	US	Doctors attention to childhood obesity	Ad hoc algorithm	External
Duran et al. [82] (2019)	1333	US	Body fat% (excess)	ANN	External

ML Method abbreviation as in Section 2.
